# Supplementary figures and images for: Different estimation methods of the modified Kies Topp-Leone model with applications and quantile regression
Source: PLoS One. 2024 Sep 13;19(9):e0307391. doi: 10.1371/journal.pone.0307391 (PMC11398704; doi:10.1371/journal.pone.0307391)

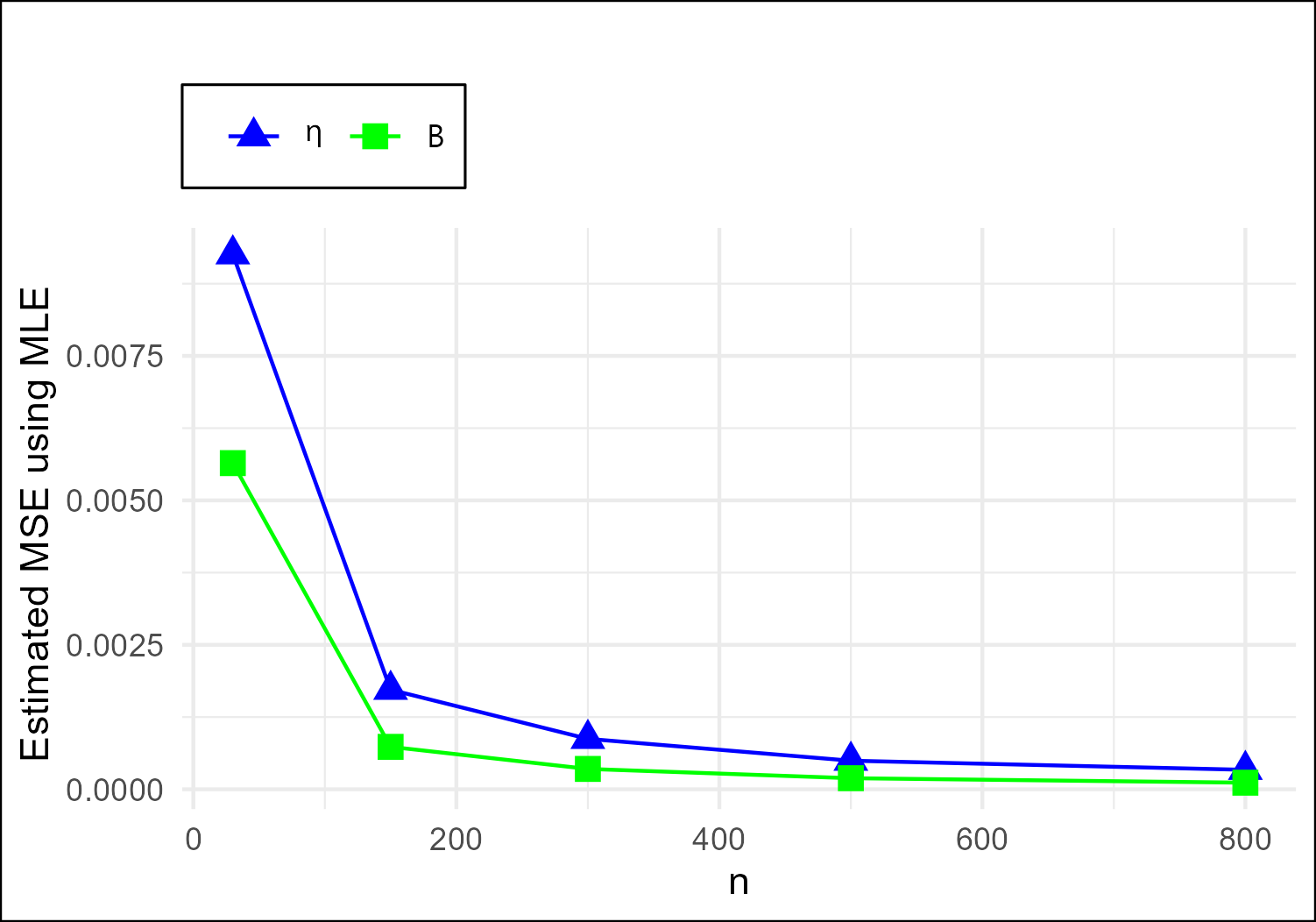

Supplement: S1 File — (ZIP) [file pone.0307391.s001.zip › Fig5a.png]

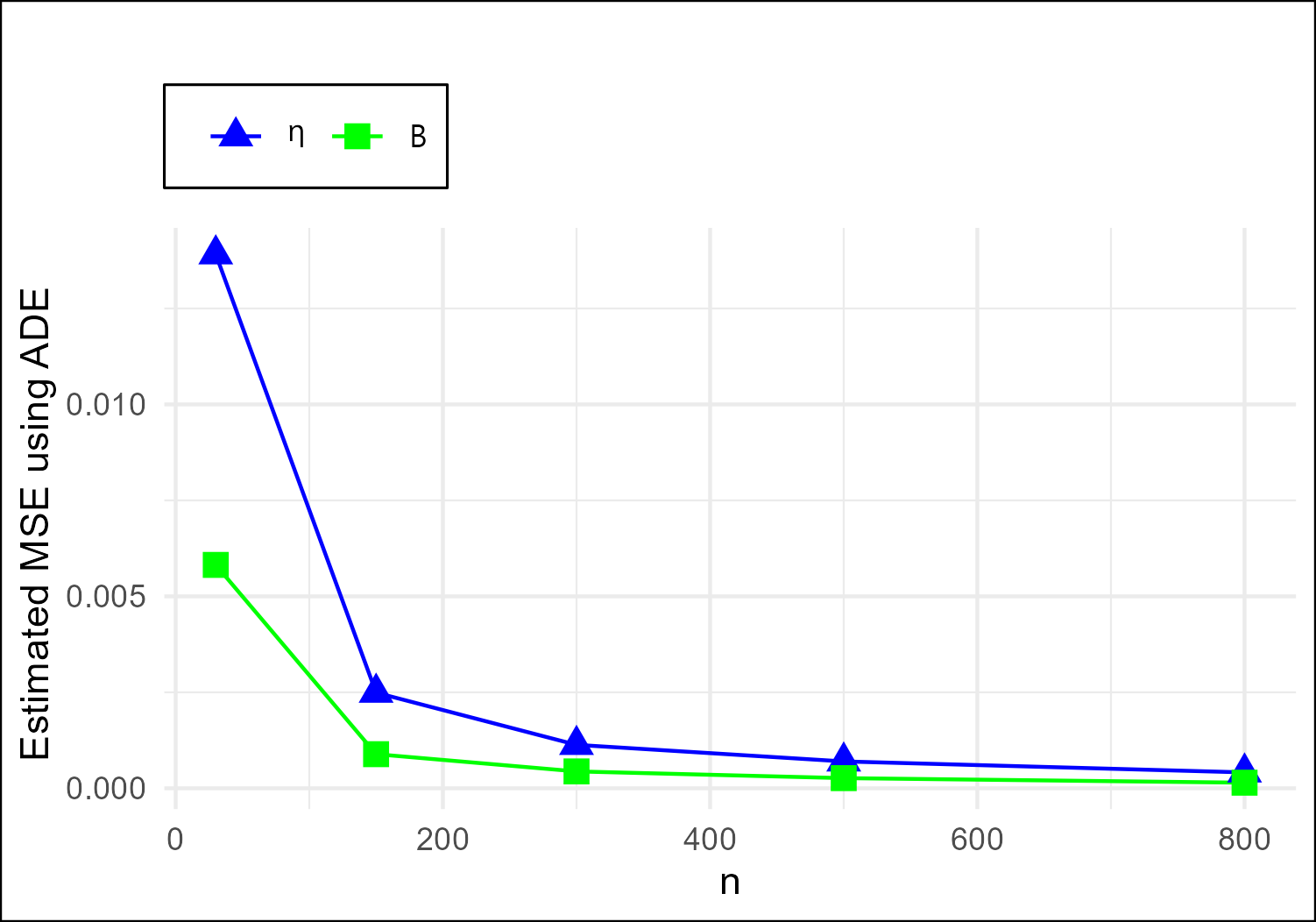

Supplement: S1 File — (ZIP) [file pone.0307391.s001.zip › Fig5b.png]

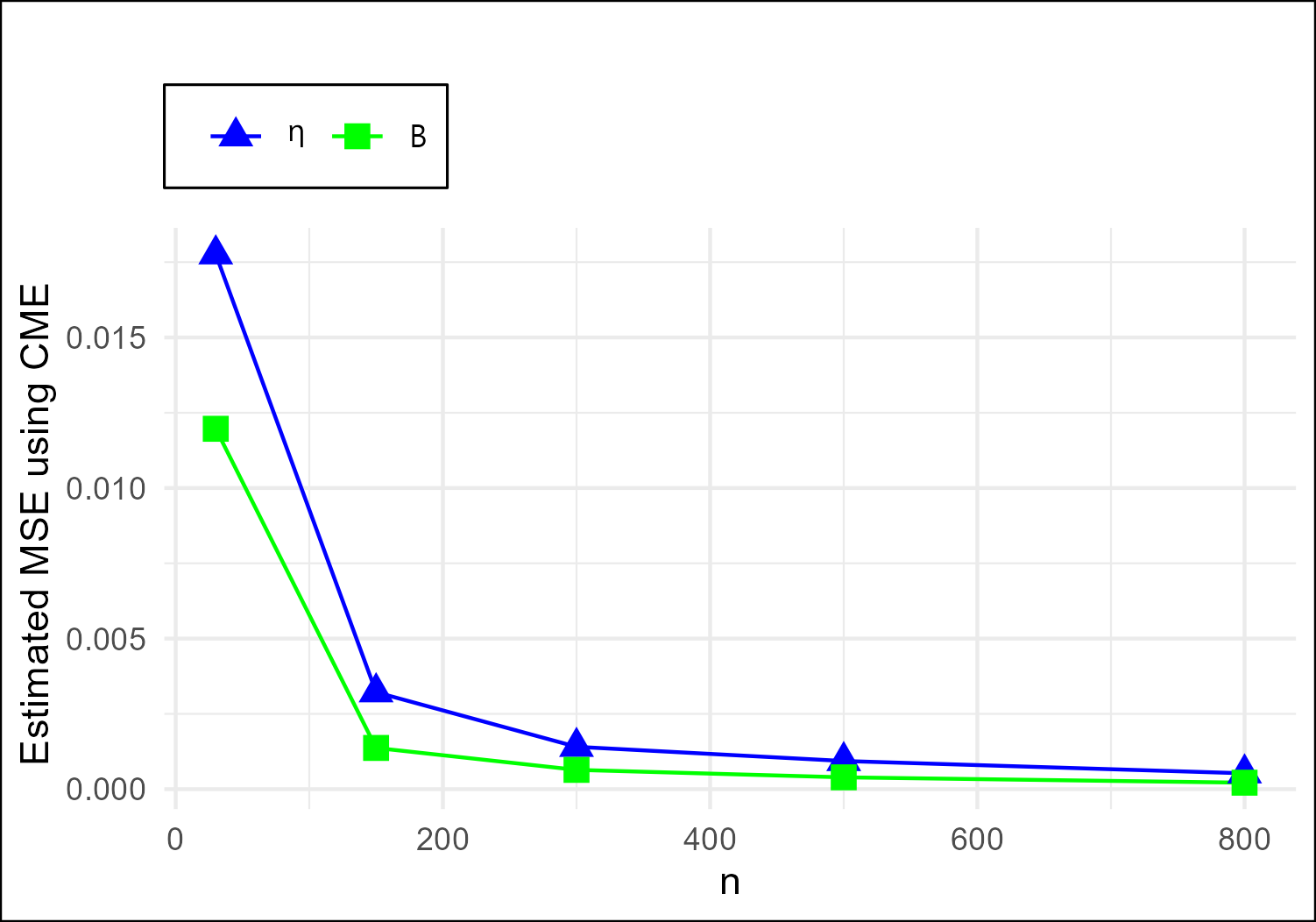

Supplement: S1 File — (ZIP) [file pone.0307391.s001.zip › Fig5c.png]

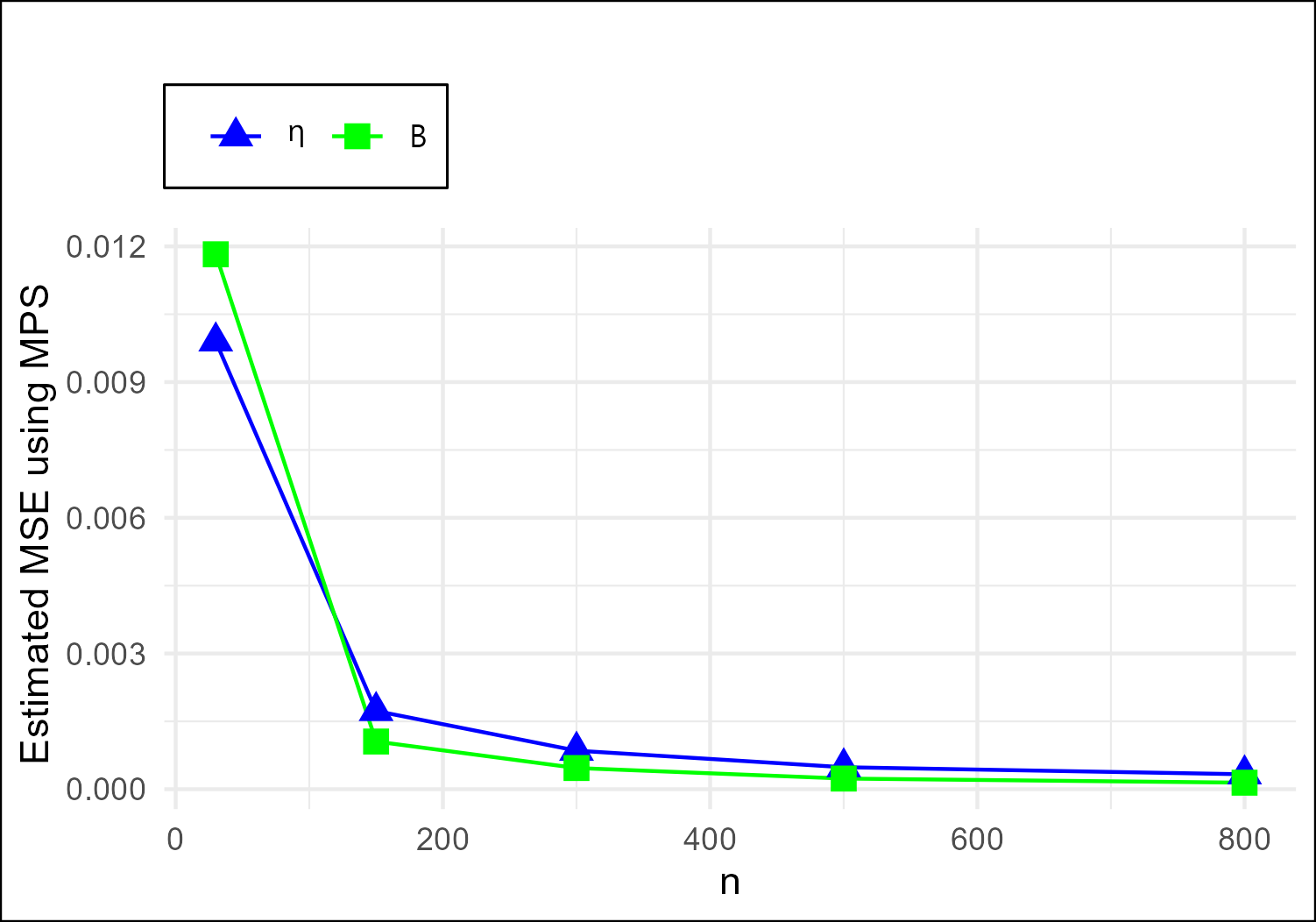

Supplement: S1 File — (ZIP) [file pone.0307391.s001.zip › Fig5d.png]

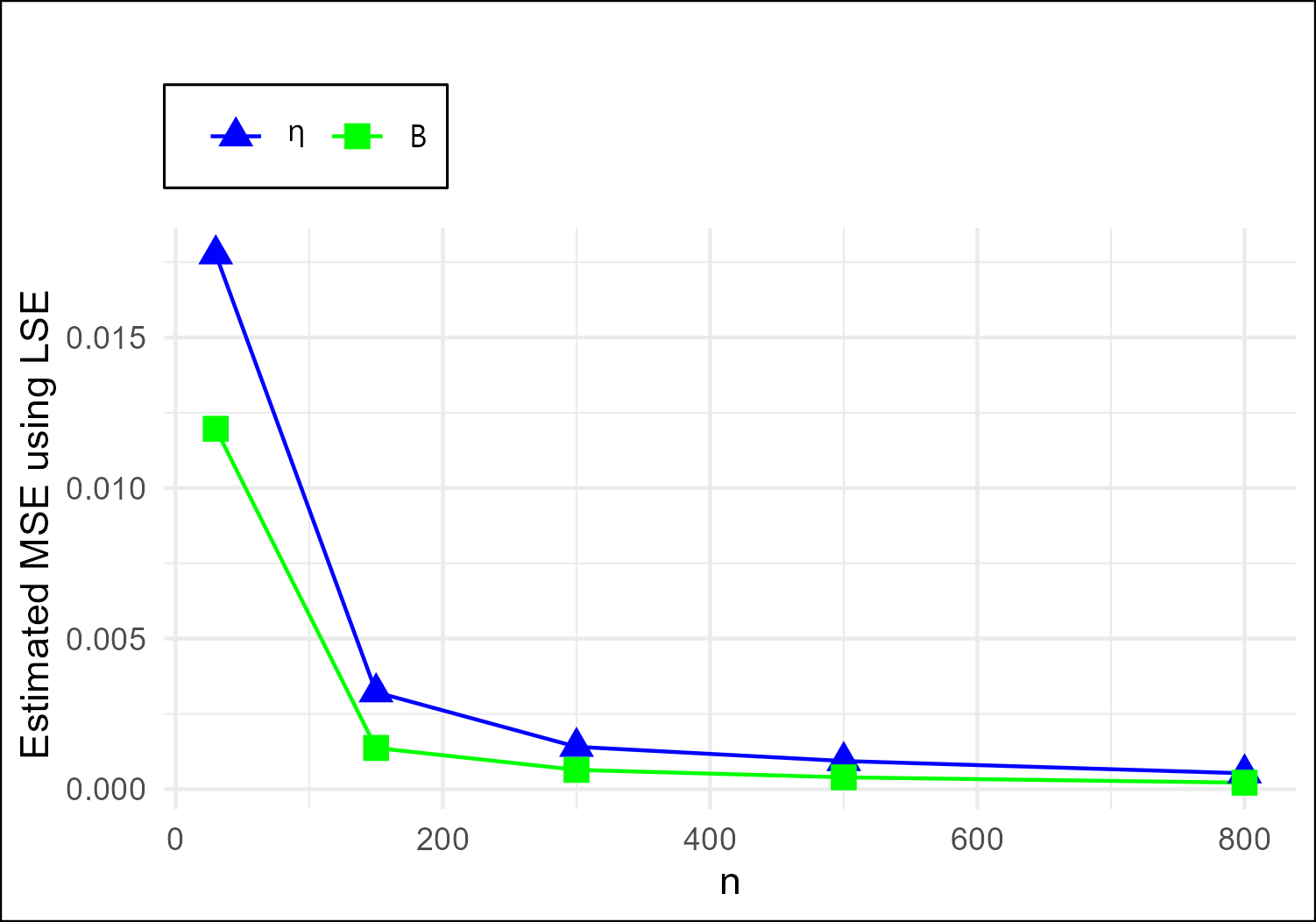

Supplement: S1 File — (ZIP) [file pone.0307391.s001.zip › Fig6a.png]

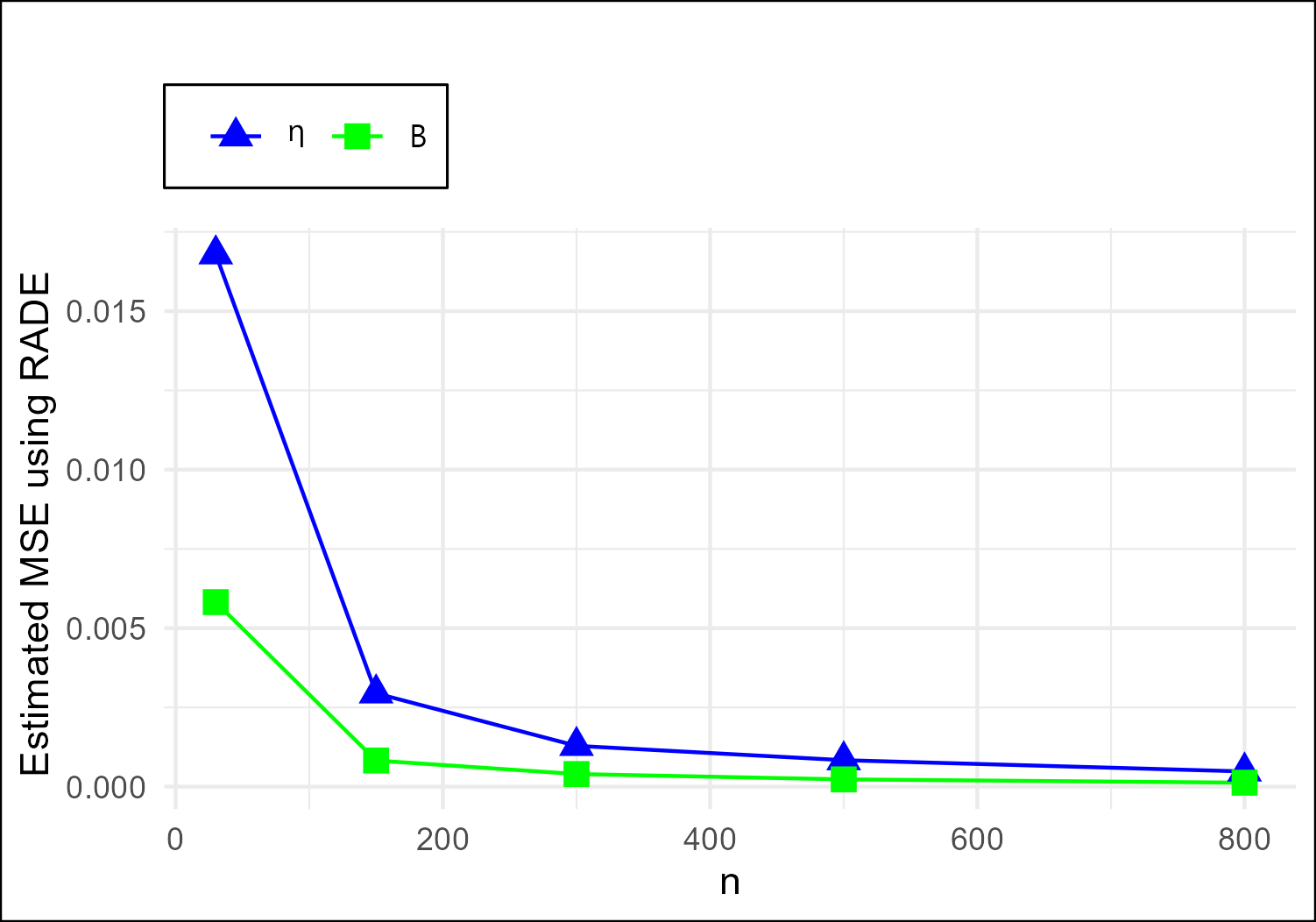

Supplement: S1 File — (ZIP) [file pone.0307391.s001.zip › Fig6b.png]

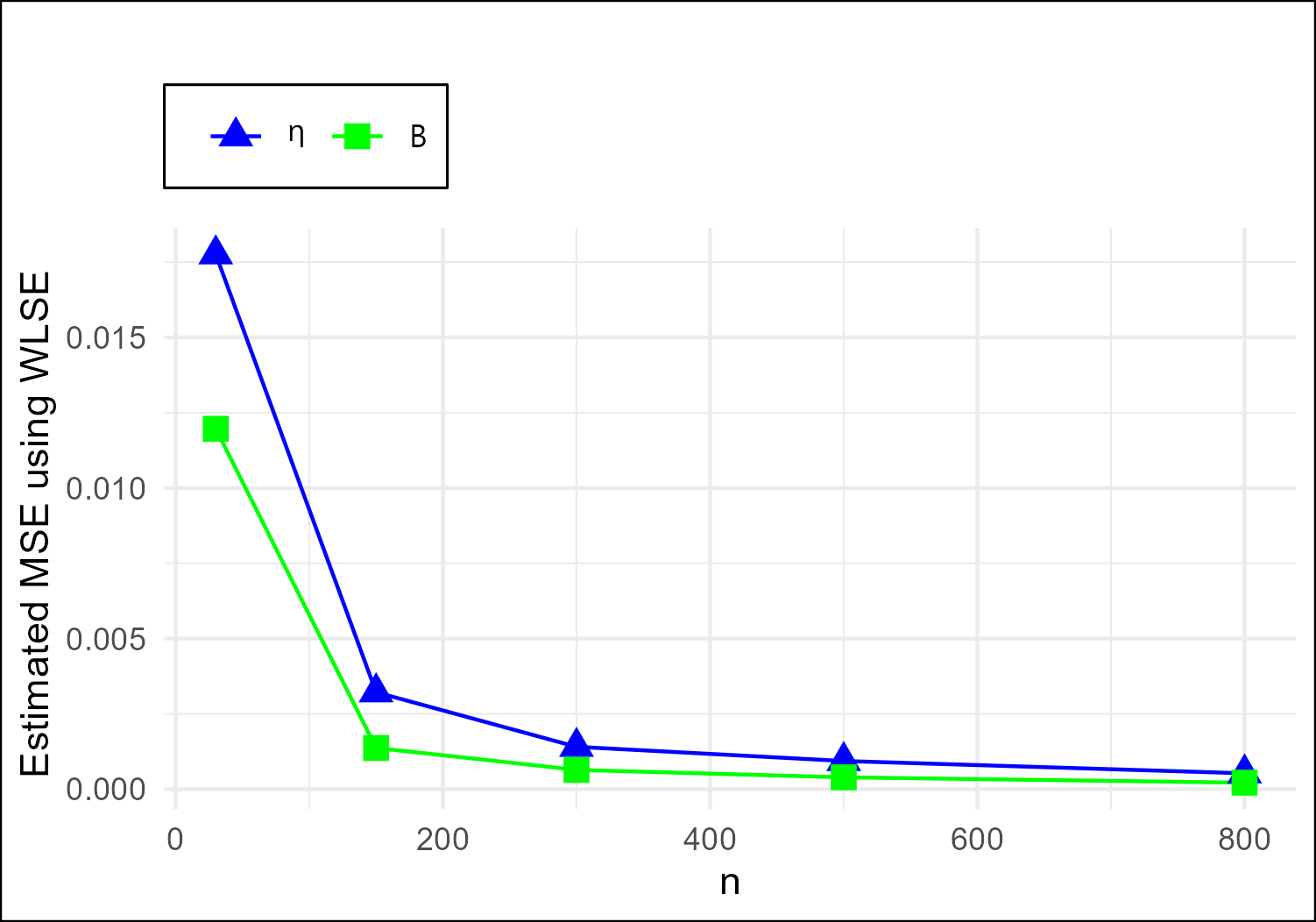

Supplement: S1 File — (ZIP) [file pone.0307391.s001.zip › Fig6c.png]

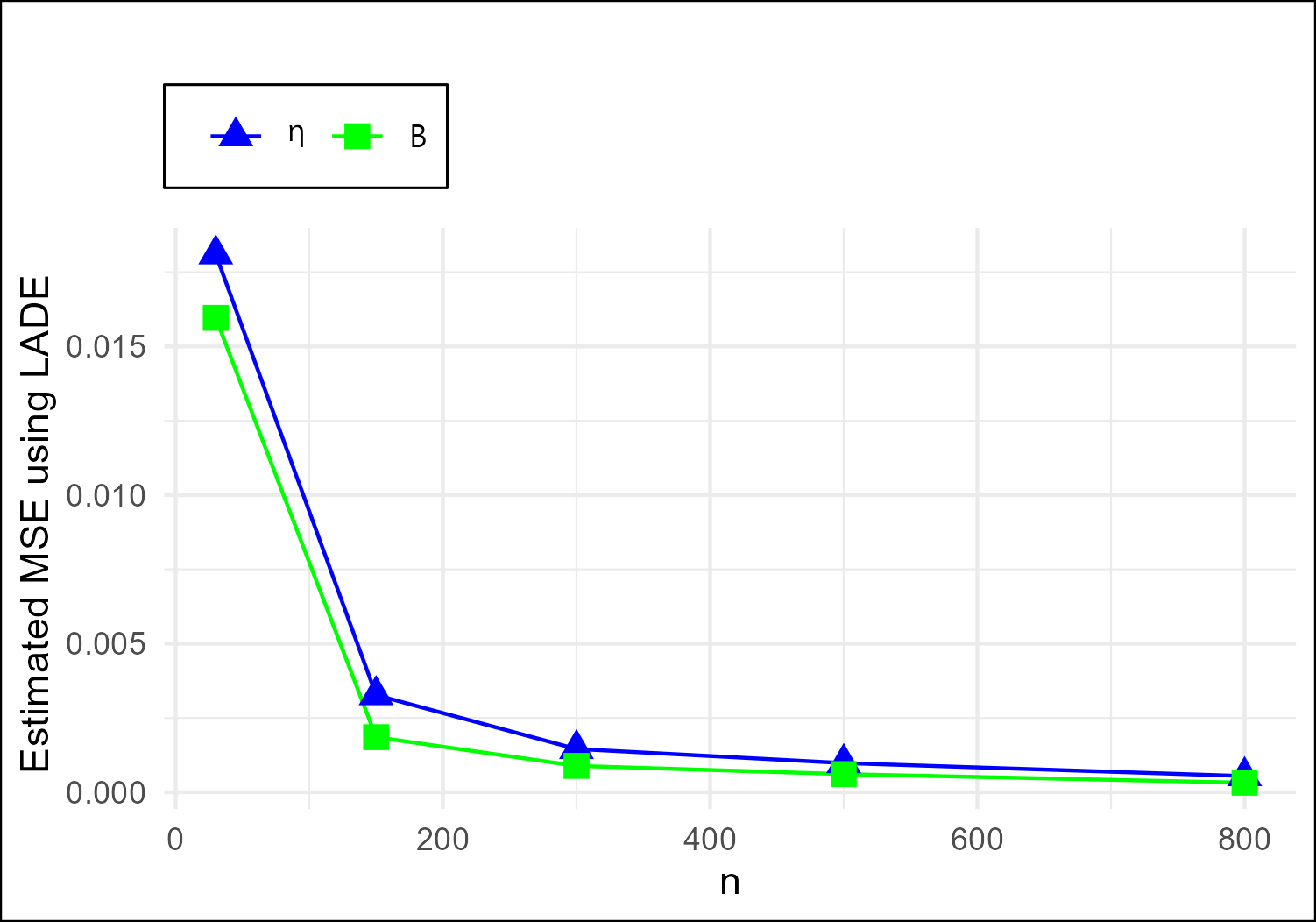

Supplement: S1 File — (ZIP) [file pone.0307391.s001.zip › Fig6d.png]

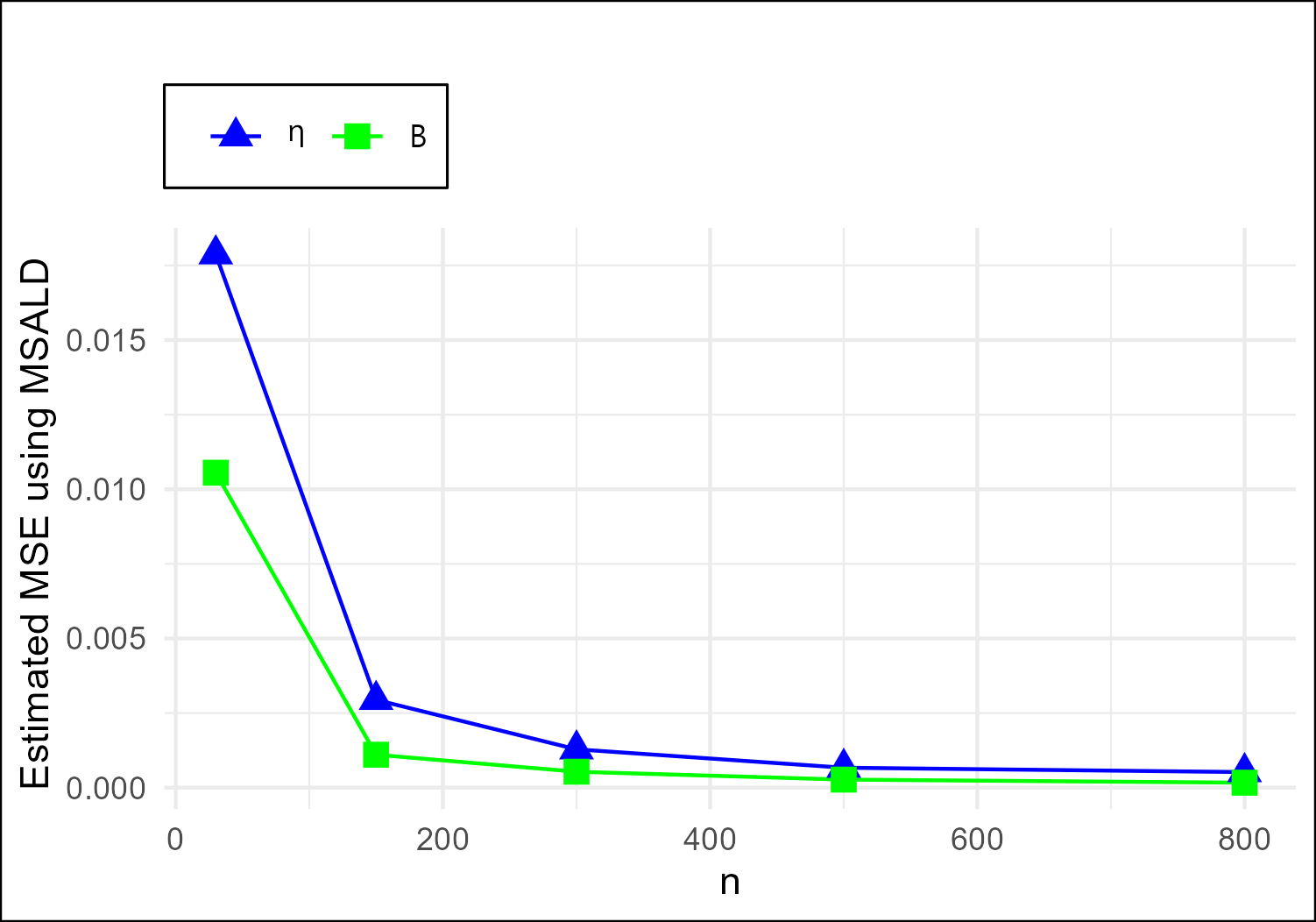

Supplement: S1 File — (ZIP) [file pone.0307391.s001.zip › Fig7a.png]

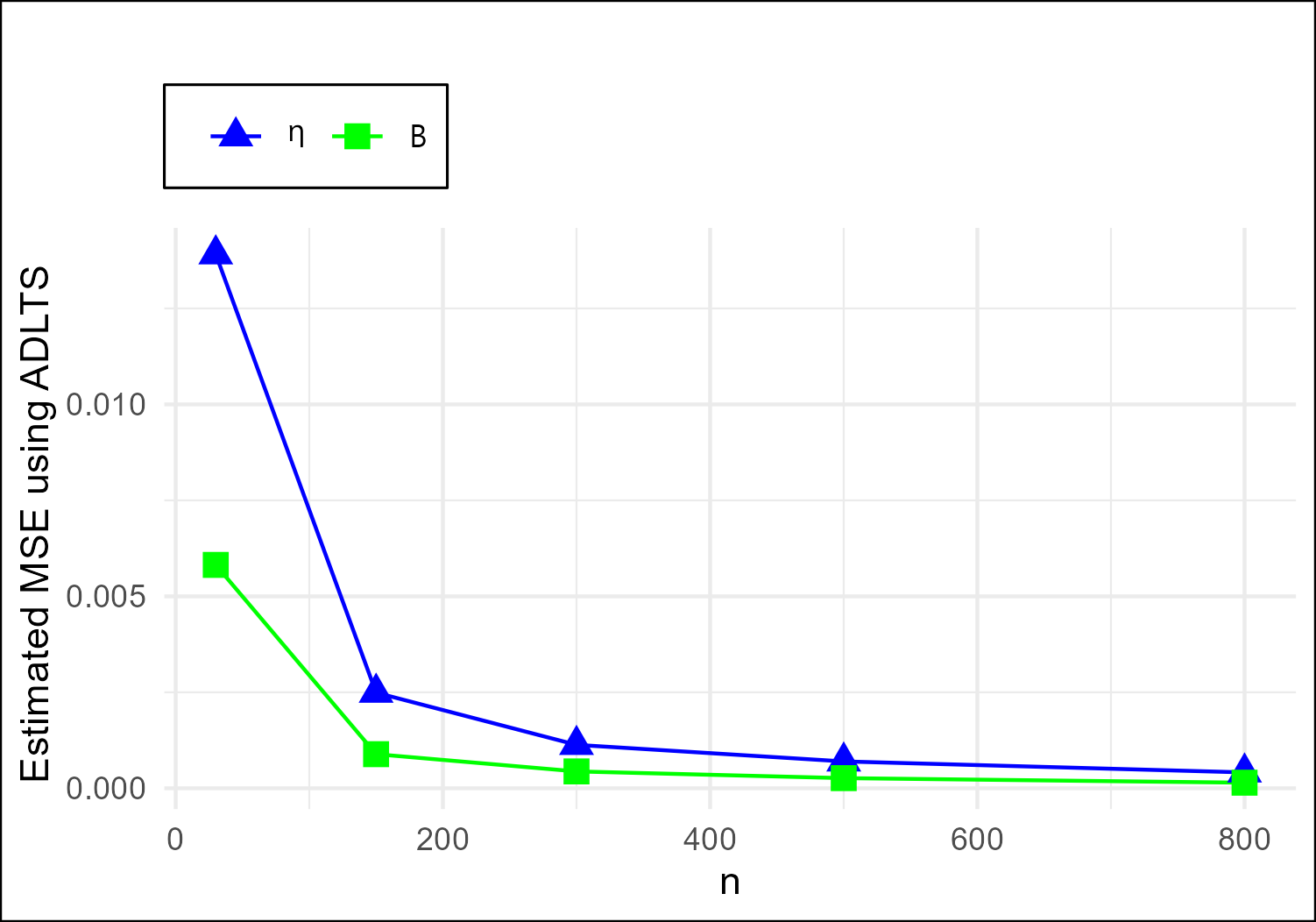

Supplement: S1 File — (ZIP) [file pone.0307391.s001.zip › Fig7b.png]

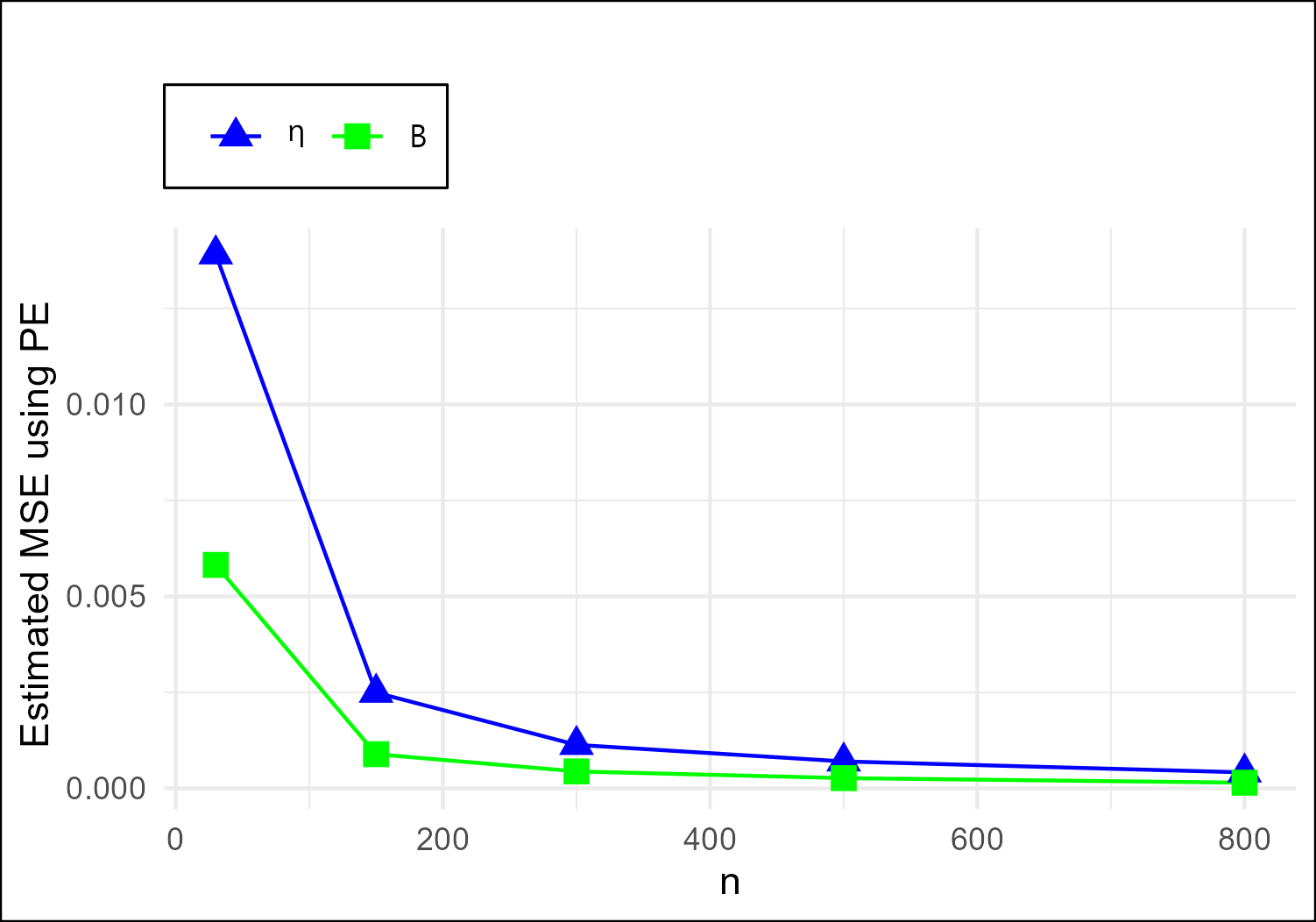

Supplement: S1 File — (ZIP) [file pone.0307391.s001.zip › Fig7c.png]

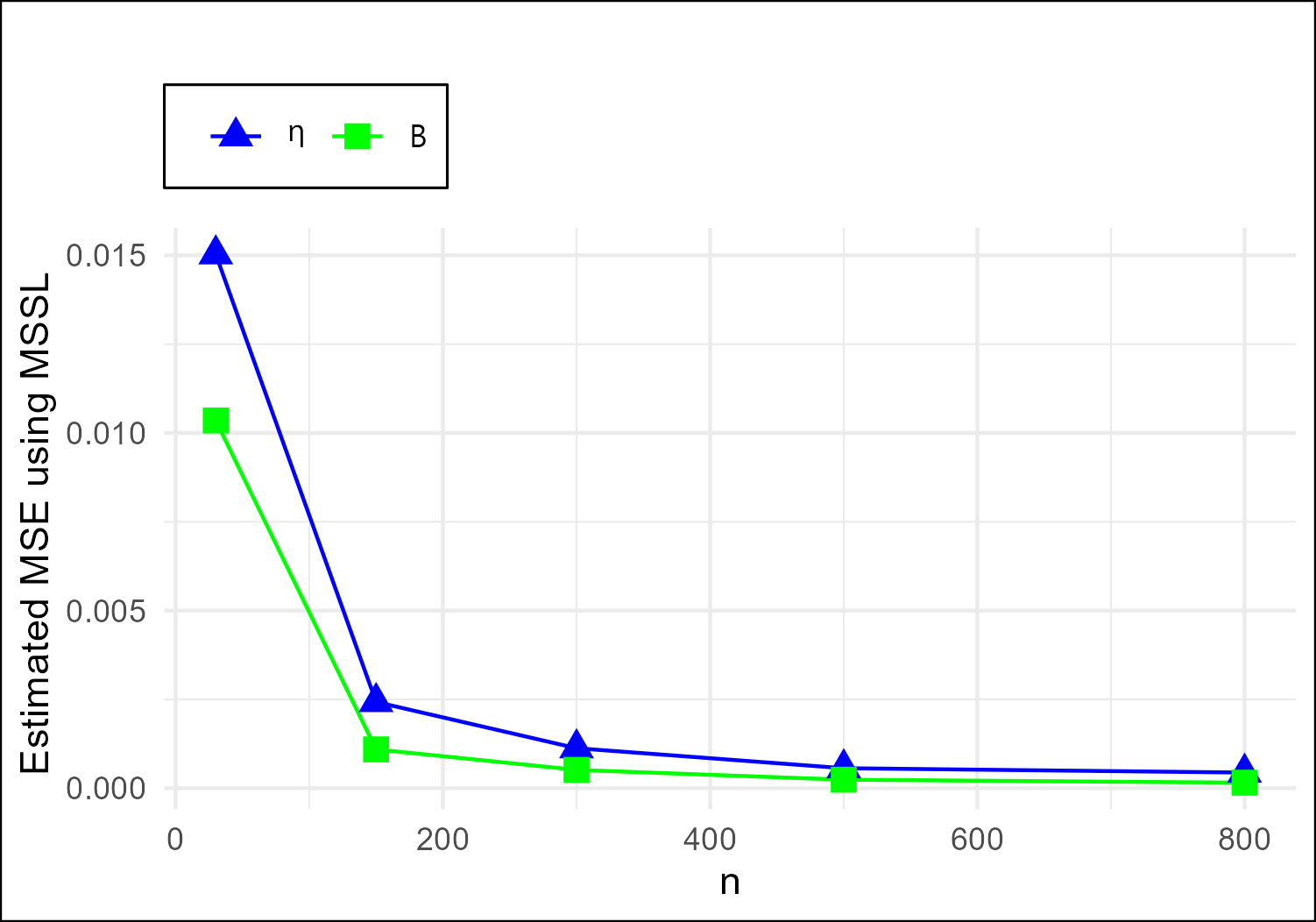

Supplement: S1 File — (ZIP) [file pone.0307391.s001.zip › Fig7d.png]

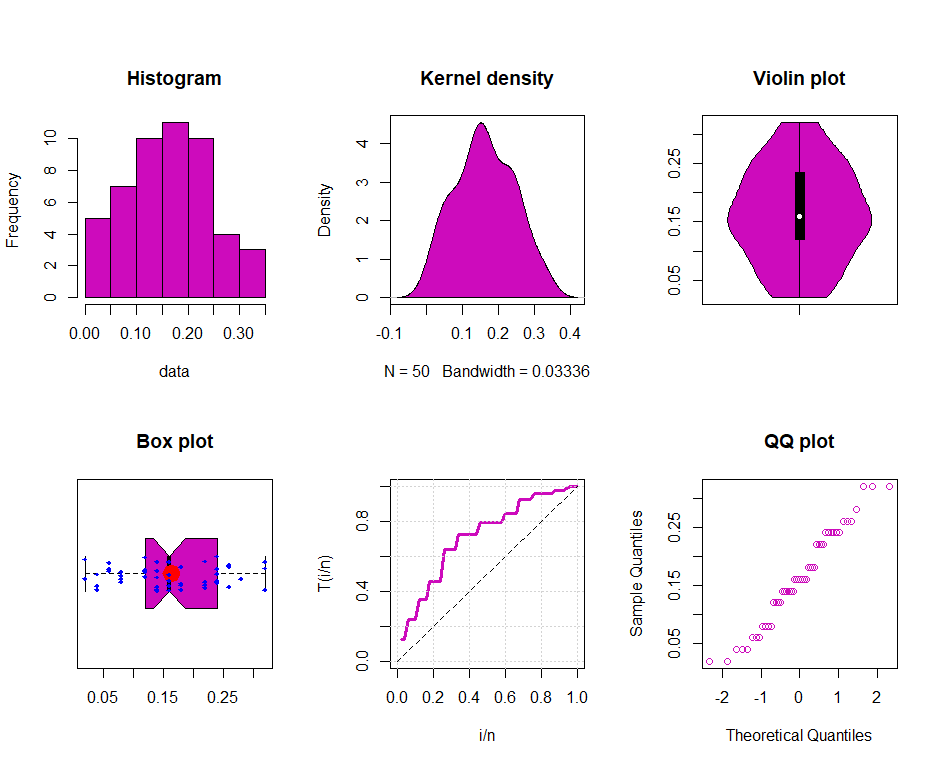

Supplement: S1 File — (ZIP) [file pone.0307391.s001.zip › Fig8.png]

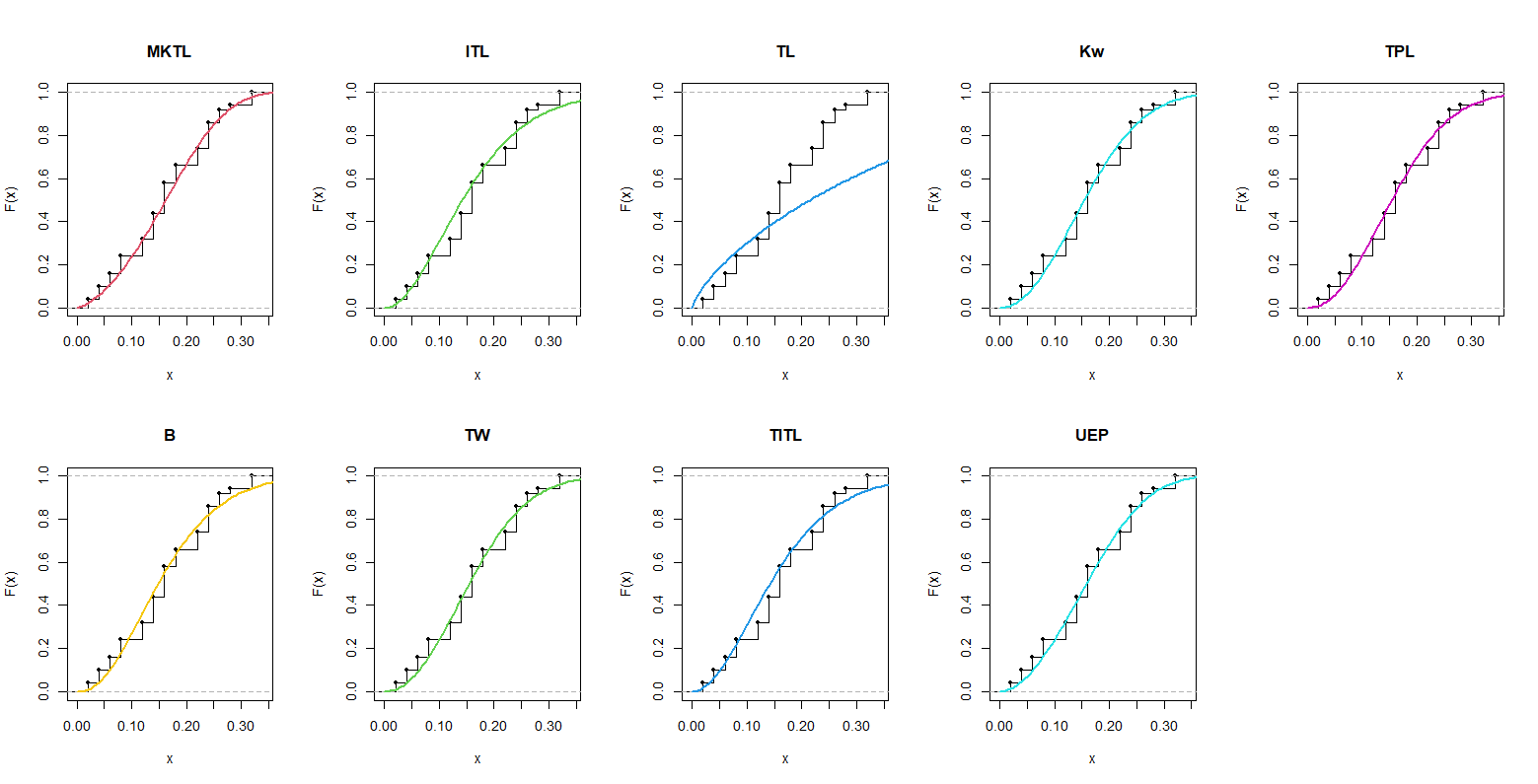

Supplement: S1 File — (ZIP) [file pone.0307391.s001.zip › Fig12.png]

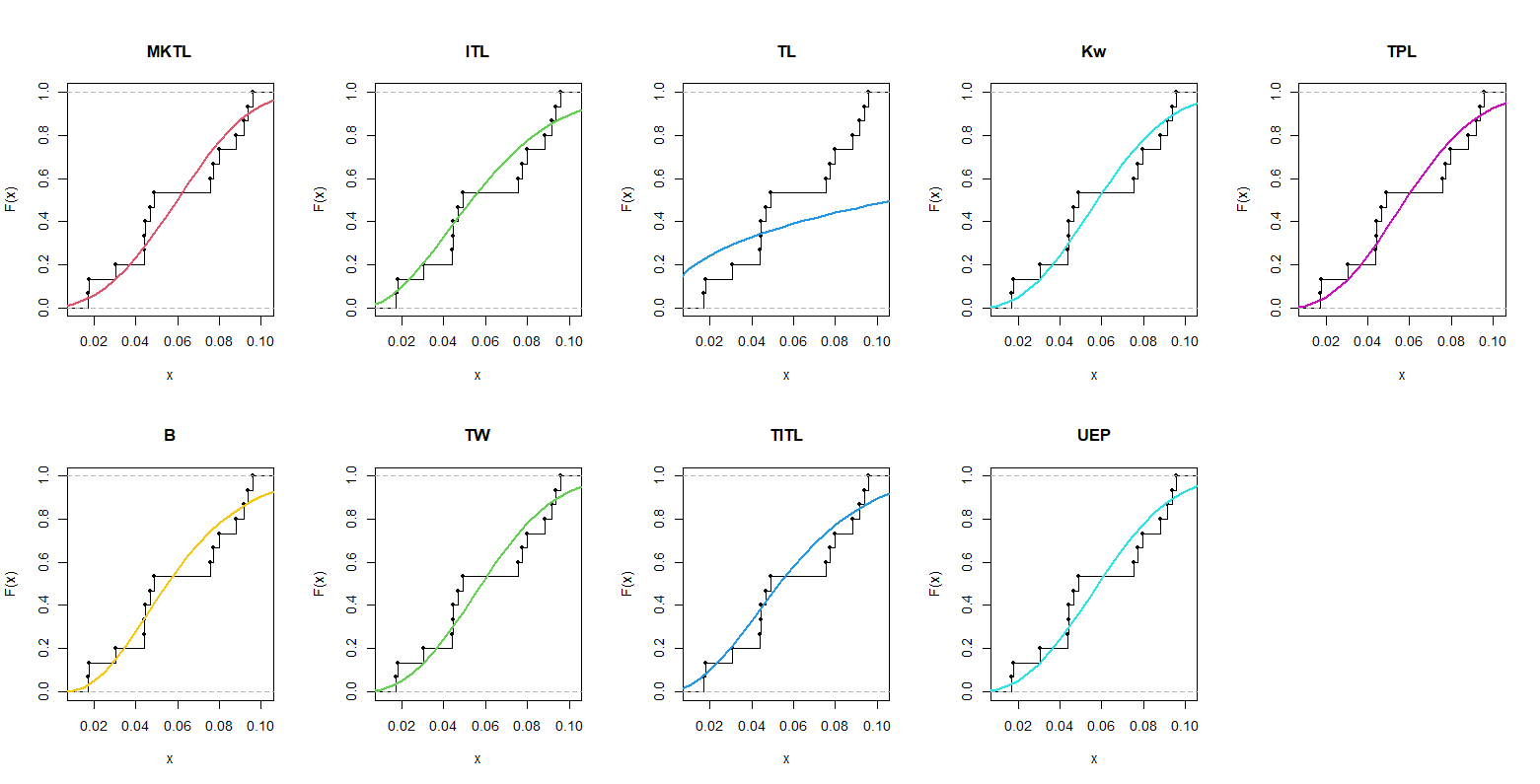

Supplement: S1 File — (ZIP) [file pone.0307391.s001.zip › Fig15.png]

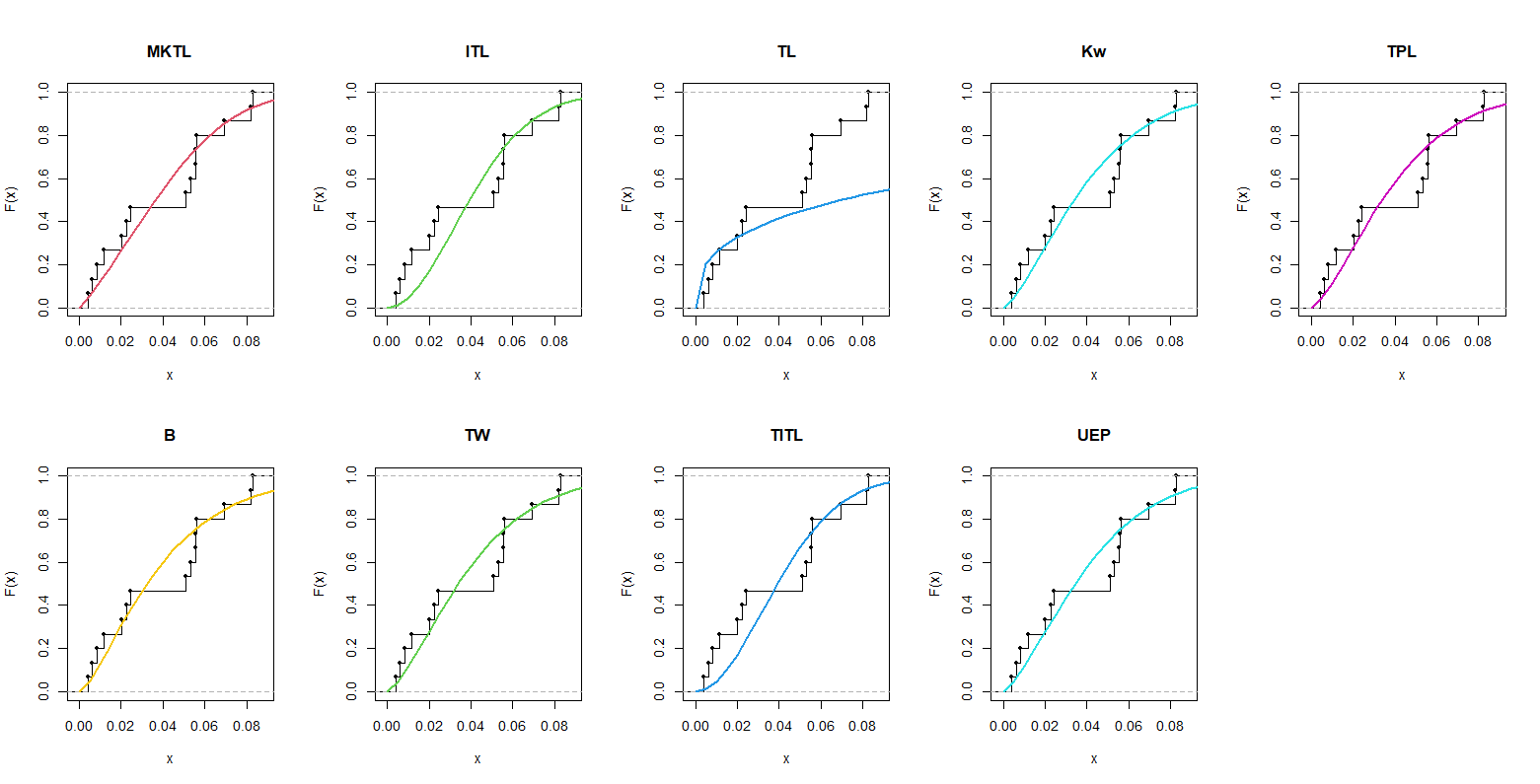

Supplement: S1 File — (ZIP) [file pone.0307391.s001.zip › Fig18.png]

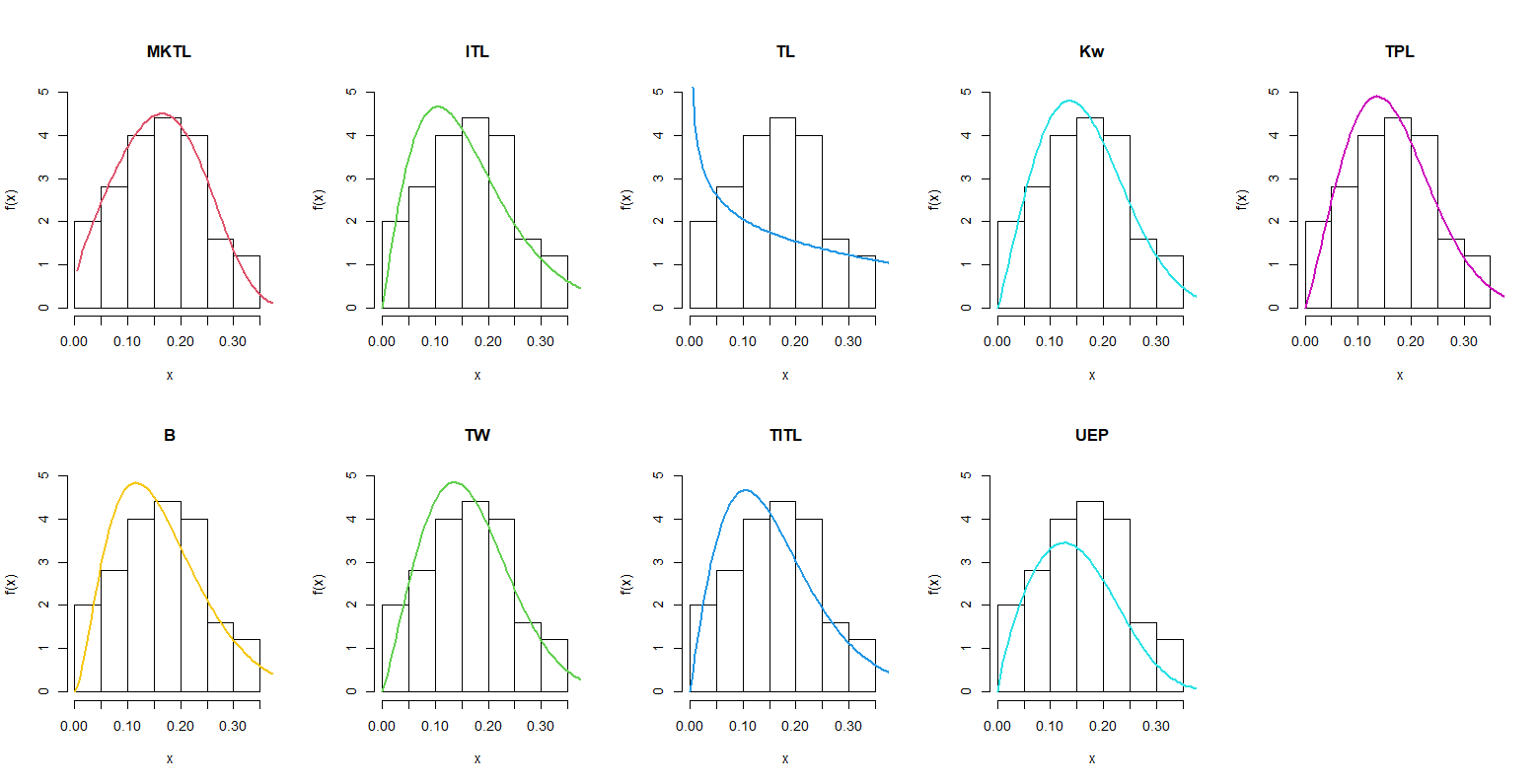

Supplement: S1 File — (ZIP) [file pone.0307391.s001.zip › Fig11.png]

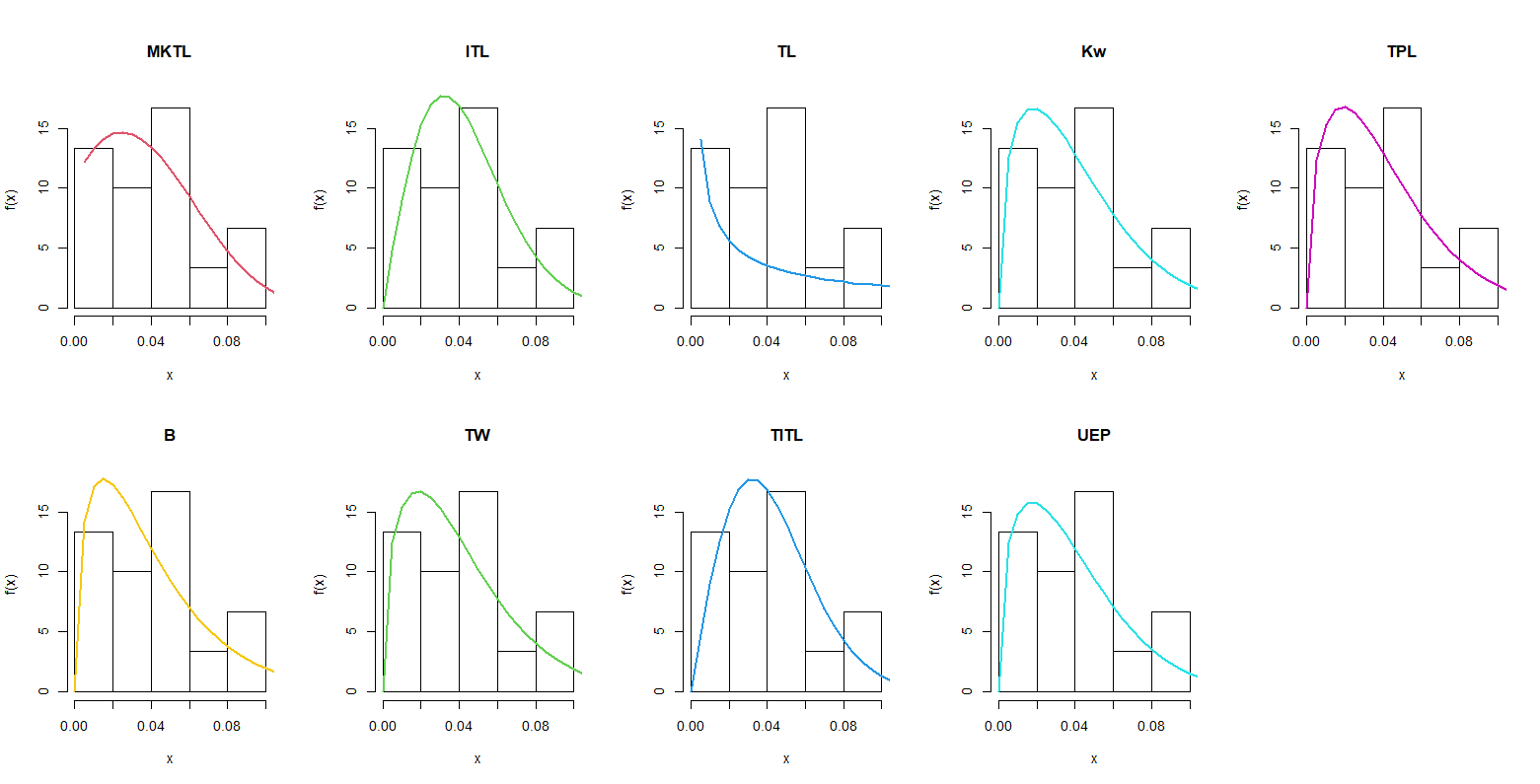

Supplement: S1 File — (ZIP) [file pone.0307391.s001.zip › Fig17.png]

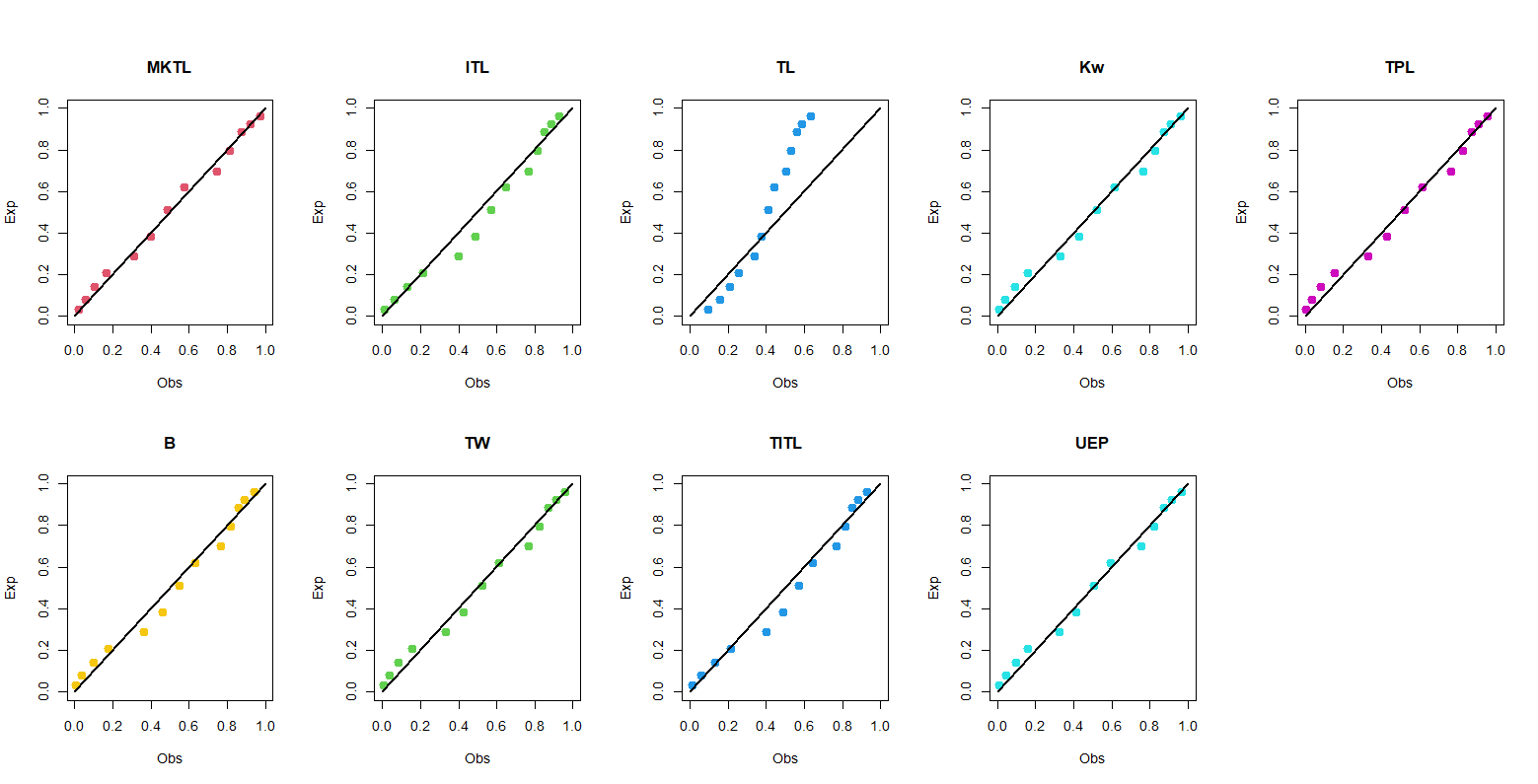

Supplement: S1 File — (ZIP) [file pone.0307391.s001.zip › Fig13.png]

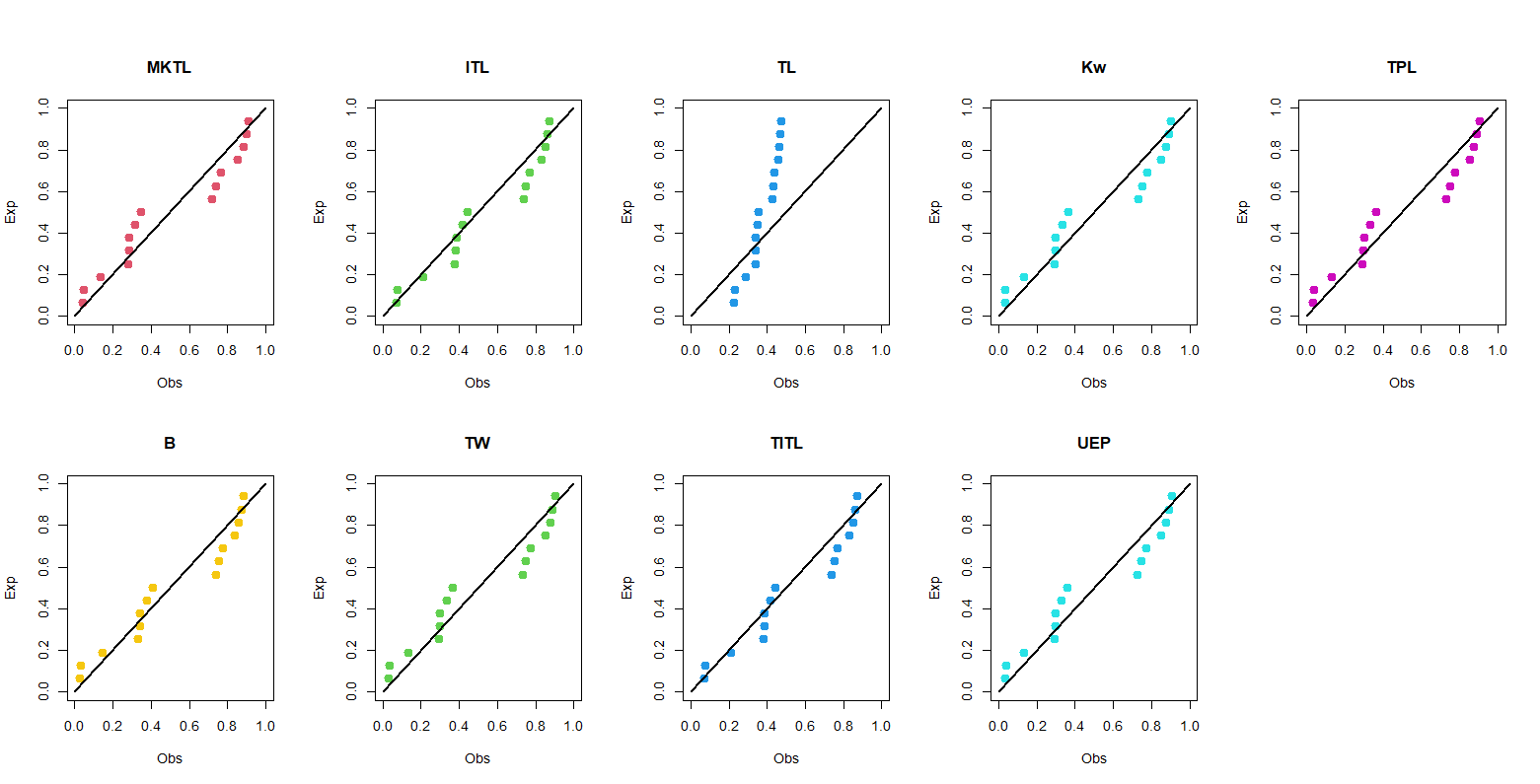

Supplement: S1 File — (ZIP) [file pone.0307391.s001.zip › Fig16.png]

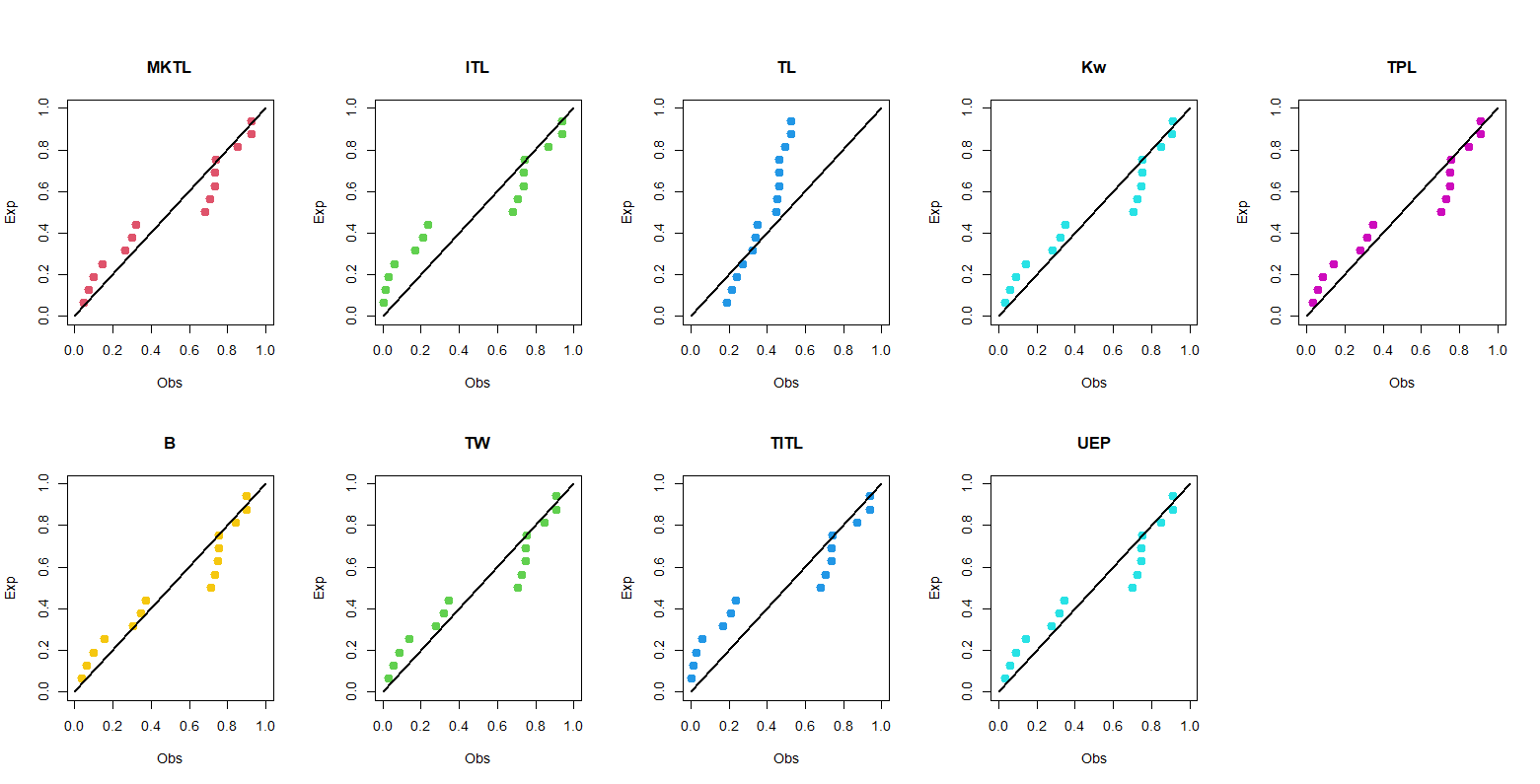

Supplement: S1 File — (ZIP) [file pone.0307391.s001.zip › Fig19.png]

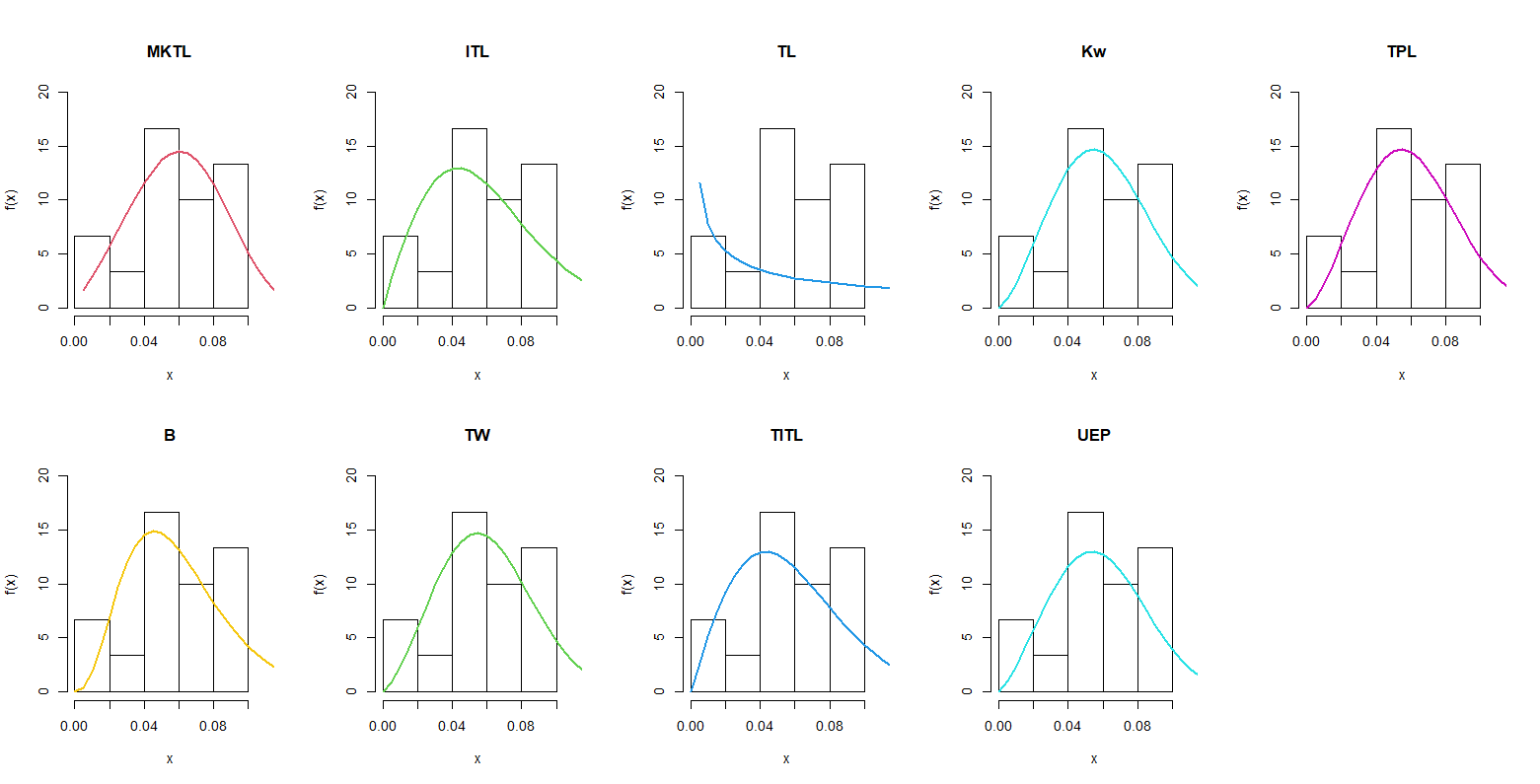

Supplement: S1 File — (ZIP) [file pone.0307391.s001.zip › Fig14.png]

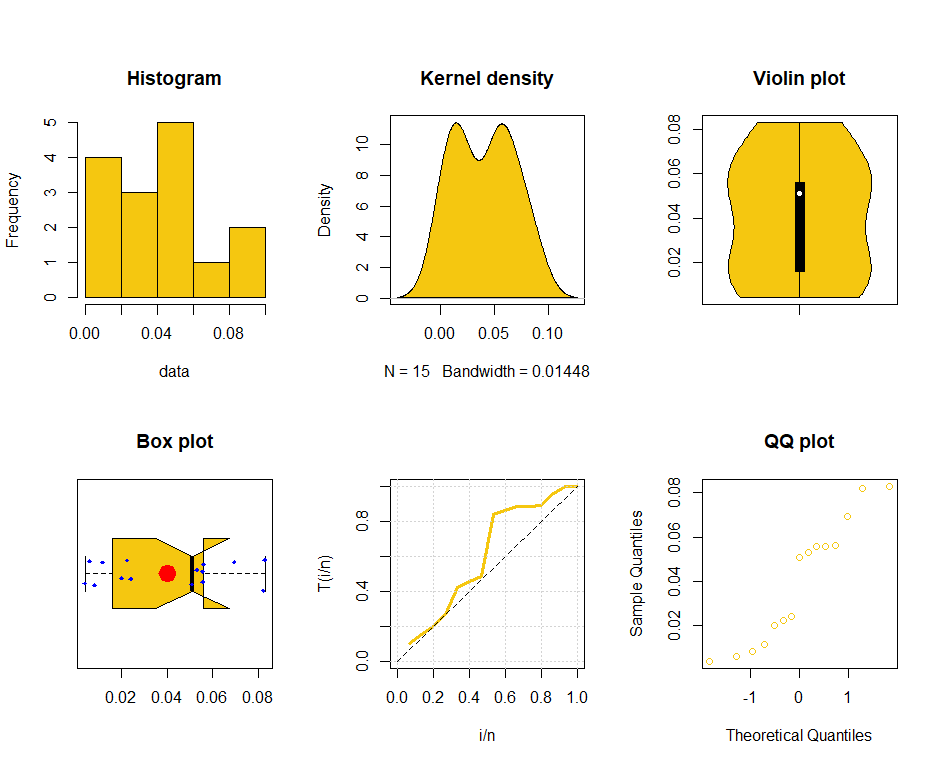

Supplement: S1 File — (ZIP) [file pone.0307391.s001.zip › Fig9.png]

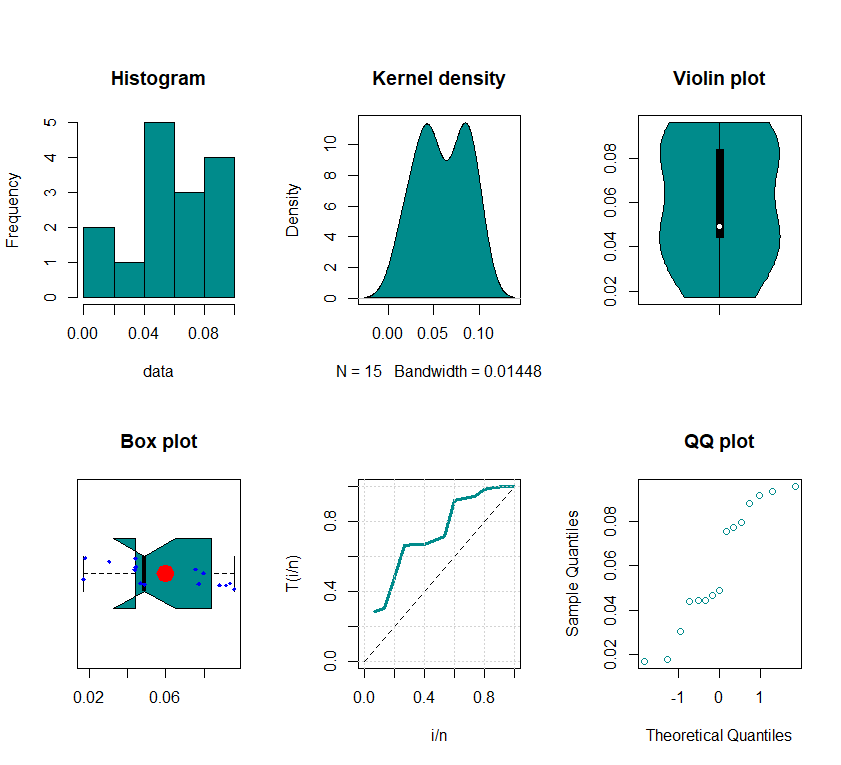

Supplement: S1 File — (ZIP) [file pone.0307391.s001.zip › Fig10.png]
