# Supplementary material for: Different estimation methods of the modified Kies Topp-Leone model with applications and quantile regression
Source: PLoS One. 2024 Sep 13;19(9):e0307391. doi: 10.1371/journal.pone.0307391 (PMC11398704; doi:10.1371/journal.pone.0307391)
Supplement: Attachment — Submitted filename: Response to the reviewers comments.docx [file pone.0307391.s003.docx]

**Response to the reviewer’s comments**

PONE-D-24-17754

Title: Different Estimation Methods of the Modified Kies Topp-Leone Model with Applications and Quantile Regression

PLOS ONE

**Dear Editors and Reviewers:**

First, the authors would like to thank the Editor in Chief, Associate editor, Lead editor, Academic editor and Anonymous referees for spending their time on the manuscript carefully. The comments of the editors and reviewers are valuable. We have taken all the suggestions/comments positively and did our best to incorporate all these suggestions in the revised version. Our point wise responses to the reviewer’s comments/suggestions are given below.

**Response to Editor**

Dear Sir/Mam, we appreciate your time in handling our paper and providing suggestions for improvement. We believe the quality of the revised version has considerably improved and hope that you find the revised manuscript satisfactory this time.

**Reply to the Reviewer #1**

Reviewer #1: This paper introduces the modified Kies Topp-Leone (MKTL) distribution for modeling data on the (0, 1) or [0, 1] interval. The shapes of the density and hazard rate functions manifest desirable shapes, making the MKTL distribution suitable for modeling data with different characteristics at the unit interval. Twelve different estimation methods are utilized to estimate the distribution parameters, and Monte Carlo simulation experiments are executed to assess the performance of the methods. The simulation results suggest that the maximum likelihood method is the superior method. The usefulness of the new distribution is illustrated by utilizing three data sets. This work is quite good, scientifically sound and suitable for publication. Therefore it must be accepted for publication.

Answer: Many thanks for you positive report.

**Reply to the Reviewer #2**

Reviewer #2: REVIEWER REPORT ON PONE-D-24-17754

TOPIC: DIFFERENT ESTIMATION METHODS OF THE MODIFIED KIES TOPPLEONE MODEL WITH APPLICATIONS AND QUANTILE REGRESSION

After carefully reviewing the manuscript of the authors, I can say they have done some good

work. However, I have the following comments which are MAJOR for the authors to address.

1. Authors did not define what Q1, Q2, Q3, BSK and MKUR measures represents in Table 1

Answer: Many thanks for this nice comment. We defined it in the revised version.

2. Authors should also provide 3D plots for the BSK and MKUR measures.

Answer: Many thanks for this nice comment. We added it in Figures 2 and 3.

3. In section 4.3, Cramer_von_Mises should be written as Cramér-von Mises.

Answer: Many thanks for this nice comment. We corrected it.

4. The word burr in the second paragraph of Section 6 should be written as Burr.

Answer: Many thanks for this nice comment. We corrected it.

5. The sum in the last column in Table 12 is not necessary. Authors should delete it.

Answer: Many thanks for this nice comment. We deleted it.

6. In Table 17, what does NaN represents. Is it an indication that the optimization did not converge for the MOKw distribution. Authors should crosscheck and address the issue.

Answer: Many thanks for this nice comment. We deleted it.

7. Authors need to carefully proof read the manuscript and address all grammatical errors.

Answer: Many thanks for this nice comment. We revised it.

8. **Add R codes with numerical value of lower and upper quintile in Appendix for figure 19: Rate of change of parameter estimates for various quantiles**
   Answer: Many thanks for this nice comment. Please attach it

######################################### RATE OF CHANGE OF Parameter Plots#######################################

########### Plotting the rate of Change of Parameter Values Across various Quantiles #############

######## alpha0, alpha1, alpha2 and beta are the estimate of the parameters for various quantiles ###

##########lapha0, lapha1, lalpha2 and lbeta are the estimated lower confidence limit for the parameters across various quantiles##

\#### uapha0, uapha1, ualpha2 and ubeta are the estimated upper confidence limit for the parameters across various quantiles##

q<-c(0.01,0.1,0.25,0.5,0.75,0.9,0.95,0.99) ######## quantiles

alpha0<-c(1.28673937,1.64753913,1.91634265,2.27689722,2.67268642,3.0198273,3.21475942,3.54988938)##### estimates of alpha0 for respective quantiles

lalpha0<-c(0.71526609,1.15010681,1.45934317,1.8586783,2.26585706,2.58945246,2.75918486,3.03678882)##### estimates of lalpha0 for respective quantiles

ualpha0<-c(1.85821265,2.14497145,2.37334213,2.69511614,3.07951578,3.45020214,3.67033398,4.06298994)##### estimates of ualpha0 for respective quantiles

alpha1<-c(-0.22829258,-0.1873418-0.164946,-0.14159633,-0.1224503,-0.11038132,-0.10525902,-0.09865827)##### estimates of alpha1 for respective quantiles

lalpha1<-c(-0.36172742,-0.29541816,-0.25920828,-0.22090185,-0.1885807,-0.16765252,-0.15866706,-0.14704675)##### estimates of lalpha1 for respective quantiles

ualpha1<-c(-0.09485774,-0.07926544,-0.07068372,-0.06229081,-0.0563199,-0.05311012,-0.05185098,-0.05026979)##### estimates of ualpha1 for respective quantiles

alpha2<-c(-0.05742771,-0.04947872,-0.04561576,-0.04194493,-0.03910326,-0.03732533,-0.03655452,-0.03555266)##### estimates of alpha2 for respective quantiles

lalpha2<-c(-0.11122383,-0.092977,-0.08429636,-0.07624885,-0.07029078,-0.06673905,-0.06525088,-0.06335722)##### estimates of lalpha2 for respective quantiles

ualpha2<-c(-0.00363159,-0.00598044-0.00693516,-0.00764101,-0.00791574,-0.00791161,-0.00785816,-0.0077481)##### estimates of ualpha2 for respective quantiles

beta<-c(0.59333507,0.59459697,0.5942175,0.59339415,0.59295771,0.59248447,0.59204514,0.59096904)##### estimates of beta for respective quantiles

lbeta<-c(0.44437311,0.44632689,0.44637274,0.44588847,0.44579111,0.44553543,0.44515686,0.44409252)##### estimates of lbeta for respective quantiles

ubeta<-c(0.74229703,0.74286705,0.74206226,0.74089983,0.74012431,0.73943351,0.73893342,0.73784556)##### estimates of ubeta for respective quantiles

windows(height=10,width=20)

par(mfrow=c(2,2))

plot(q,alpha0,type="l",xlab="Quantile Level",ylab=expression(paste(hat(alpha[0]))),ylim=c(0,5))

lines(q,lalpha0,type="l",col="red")

lines(q,ualpha0,type="l",col="red")

legend("bottomright",legend=c("Point Estimate","95% CI."),col=c("black","darkgreen"),lty=1,text.font=1,cex=0.6,inset=c(0.1,0.1))

\# Fill area between lines

polygon(c(q, rev(q)), c(alpha0, rev(lalpha0)), col = "darkgreen")

polygon(c(q, rev(q)), c(alpha0, rev(ualpha0)), col = "darkgreen")

#################

plot(q,alpha1,type="l",xlab="Quantile Level",ylab=expression(paste(hat(alpha[1]))),ylim=c(-0.5,0))

lines(q,lalpha1,type="l",col="red")

lines(q,ualpha1,type="l",col="red")

legend("bottomleft",legend=c("Point Estimate","95% CI"),col=c("black","darkgreen"),lty=1,text.font=1,cex=0.6,inset=c(0.1,0.1))

polygon(c(q, rev(q)), c(alpha1, rev(lalpha1)), col = "darkgreen")

polygon(c(q, rev(q)), c(alpha1, rev(ualpha1)), col = "darkgreen")

##############################

plot(q,alpha2,type="l",xlab="Quantile Level",ylab=expression(paste(hat(alpha[2]))),ylim=c(-0.6,0.1))

lines(q,lalpha1,type="l",col="red")

lines(q,ualpha2,type="l",col="red")

legend("bottomright",legend=c("Point Estimate","95% CI"),col=c("black","darkgreen"),lty=1,text.font=1,cex=0.6,inset=c(0.1,0.1))

polygon(c(q, rev(q)), c(alpha2, rev(lalpha1)), col = "darkgreen")

polygon(c(q, rev(q)), c(alpha2, rev(ualpha2)), col = "darkgreen")

##################

plot(q,beta,type="l",xlab="Quantile Level",ylab=expression(paste(hat(beta))),ylim=c(0,0.9))

lines(q,lbeta,type="l",col="red")

lines(q,ubeta,type="l",col="red")

legend("bottomright",legend=c("Point Estimate","95% CI"),col=c("black","darkgreen"),lty=1,text.font=1,cex=0.6,inset=c(0.1,0.1))

polygon(c(q, rev(q)), c(beta, rev(lbeta)), col = "darkgreen")

polygon(c(q, rev(q)), c(beta, rev(ubeta)), col = "darkgreen")
